# Acetabular defect classification and management: Revision arthroplasty of the acetabular cup based on 3-point fixation

**DOI:** 10.1007/s00132-020-03895-8

**Published:** 2020-02-28

**Authors:** Mohamed Ghanem, Dirk Zajonz, Christoph-Eckhard Heyde, Andreas Roth

**Affiliations:** Department of Orthopaedic Surgery, Traumatology and Plastic Surgery, Universitätsklinikum Leipzig, Liebigstraße 20, 04103 Leipzig, Germany

**Keywords:** Retrospective study, Total hip replacement, Revision arthroplasty, Computed tomography, Diagnostic imaging, Retrospektive Studie, Hüfttotalendoprothese, Revisionsarthroplastik, Computertomographie, Diagnostische Bildgebung

## Abstract

**Background:**

The purpose of this study was to provide a practicable and contemporary classification system that is reliable and pragmatic with respect to perioperative evaluation, planning, scientific comparison and analysis.

**Material and methods:**

This was a retrospective study of 160 patients who underwent acetabular revision surgery after THR due to loosening of the acetabular cup. The assessment of the acetabular defect was based on intraoperative description of the bony configuration of the acetabulum as well as on standardized preoperative planning images (pelvic overview and axial view of the hip joint). Preoperative computed tomography (CT) was carried out in individual cases.

**Results:**

Acetabular bone defects were classified into 4 types based on whether or not a 3-point fixation of the acetabular cup within the boundaries of the acetabular cavity was possible. Minor segmental defects or cup loosening without bone loss can be treated with standard hemispherical acetabular components. Bone loss can be filled with bone grafts and/or treated by the appropriate acetabular component in order to ensure stable anchorage. When conventional revision cups are no longer suitable a custom made partial pelvic replacement can be used.

**Conclusion:**

The proposed classification mainly relies on intraoperative findings which were confirmed by preoperative imaging in 154 cases out of 160 (96.25%); however, meticulous preoperative planning based on X‑ray radiographs must be carried out. In addition, a CT scan must be performed whenever type III or type IV defects are anticipated. Compared to the existing classification systems, we can state that our classification system is practicable and pragmatic and simplifies the assessment of bone defects.

## Abstract (de)

**Hintergrund:**

Das Ziel dieser Studie war es, ein praktikables und zeitgemäßes Klassifikationssystem bereitzustellen, das in Bezug auf perioperative Bewertung, Planung, wissenschaftlichen Vergleich und Analyse zuverlässig und praxisorientiert ist.

**Material und Methoden:**

Es handelte sich um eine retrospektive Studie mit 160 Patienten, die sich nach einer Hüfttotalendoprothese (THR) aufgrund einer Lockerung der Hüftgelenkpfanne einer Revisionsoperation unterzogen. Die Beurteilung des Pfannendefekts basierte auf der intraoperativen Beschreibung der knöchernen Konfiguration des Azetabulums sowie auf standardisierten präoperativen Planungsbildern (Beckenübersicht und axiale Ansicht des Hüftgelenks). In einzelnen Fällen wurde eine präoperative Computertomographie (CT) durchgeführt.

**Ergebnisse:**

Azetabuläre Knochendefekte wurden in 4 Typen eingeteilt, je nachdem, ob eine 3‑Punkt-Fixation der Hüftgelenkpfanne innerhalb der Grenzen der azetabulären Kavität möglich war oder nicht. Geringfügige segmentale Defekte oder Pfannenlockerungen ohne Knochenverlust können mit halbkugelförmigen Standard-Pfannenkomponenten behandelt werden. Knochenverlust kann mit Knochentransplantaten aufgefüllt und/oder mit der entsprechenden Pfannenkomponente behandelt werden, um eine stabile Verankerung zu gewährleisten. Wenn herkömmliche Revisionspfannen nicht mehr geeignet sind, kann ein maßgeschneiderter teilweiser Beckenersatz verwendet werden.

**Schlussfolgerung:**

Die vorgeschlagene Klassifikation beruht hauptsächlich auf intraoperativen Befunden, die in 154 von 160 Fällen (96,25%) durch präoperative Bildgebung bestätigt wurden; allerdings muss eine sorgfältige präoperative Planung auf der Grundlage von Röntgenaufnahmen durchgeführt werden. Darüber hinaus muss immer dann ein CT-Scan durchgeführt werden, wenn Defekte vom Typ III oder IV zu erwarten sind. Im Vergleich zu den bestehenden Klassifikationssystemen können wir konstatieren, dass unser Klassifikationssystem praktikabel und praxisorientiert ist und die Beurteilung von Knochendefekten vereinfacht.

## Introduction

The aging population is a positive manifestation of developed and industrialized countries [18]. In these countries, old people manage to preserve general activities and sufficient mobility. The increasing number of joint replacement surgery and revision arthroplasty procedures, especially of the large joints of the lower extremities, is understandable [4, 18, 21]. According to the German Endoprosthesis Register more than 25% of revision surgeries on artificial hip joints were indicated due to loosening of the acetabular cup [4]. In other reports the acetabular component was affected twice as often as the stem [9, 21]. Revision surgery of the acetabular cup has to ensure stability of the acetabular component and restore the center of rotation of the hip joint. These prevent migration, recurrent loosening and dislocation. The results after revision surgery of hip arthroplasty are difficult to compare because of the diversity of the revision components used, the different classifications of acetabular bone defects and the insufficient documentation of the initial clinical findings [6, 7, 13, 15, 22]. There are several published classification systems for acetabular bone defects in THA [1, 3, 5, 11, 12, 19, 20]. Although similarities exist between classification systems, each one has a unique grading scale ranging from mild to severe bone defects [17]. Precise planning of the operative procedure is indispensable. The bone structure must be analyzed and classified with respect to bone loss. A preoperative AP overview of the pelvis does not enable an adequate assessment of the three-dimensional extent of the bone defects [1]. Important additional information about the anterior and posterior acetabular columns can only be obtained by means of special X‑ray techniques through the ala and obturator views, as well as in the faux profile view. Computer tomographic imaging, although limited by metal artifacts, still provides further information on the intraoperative need for special implants or bone grafts; however, it is only the intraoperative finding after the removal of the implants that provides complete clarity of the bony configuration of the acetabulum [7, 9]. A practical, reproducible and valid classification system is needed not only for preoperative planning but above all for scientific evaluations and comparisons. Some of the previously known classifications undoubtedly gained wide acceptance; however, these classifications also have weaknesses, which were described in the literature as too coarse, too confusing or too complex [1, 7, 9].

The purpose of this study was to provide a practical and contemporary classification system that is reliable and pragmatic with respect to perioperative evaluation, planning, scientific comparison and analysis.

## Material and methods

Prior to the start of the investigation, the ethics committee of the local university was consulted. After examination, a positive vote was issued. The vote-number of the audit authority is 083/19-ek.

The classification system of acetabular bone defects that is recommended is based on a retrospective study of surgical interventions performed in this department. As part of this monocentric retrospective case analysis, patients were identified in this clinic from January 2009 to December 2018 who had received acetabular revision surgery after THR due to loosening of the acetabular cup. This was done by computerized searching for the ICD 10 diagnostic keys and operative reports in the hospital’s own documentation software (SAP AG, Walldorf, Germany). The study group included patients with indications for acetabular revision surgery due to loosening of the acetabular cup after THR identified independent of the study. A written consent was documented in all cases. In order to have a homogeneous collective of patients for this study, patients with septic loosening where explantation of the endoprosthetic components was carried out and reimplantation was performed in a further setting were excluded. Accordingly, 188 cases were identified. Furthermore, 28 cases in which the operative report did not provide an exact description of the bony configuration of the acetabulum were also excluded. Accordingly, a total of 160 patients, 69 males and 91 females, could be identified for the corresponding period. The patients were treated with implants according to the bony configuration of the acetabular cup. Implants from the following companies were used: AQ Implants (Ahrensburg, Germany), currently AQ Solutions GmbH (Hürth, Germany), DePuy-Synthes (Part of the Johnson & Johnson Family of Companies, USA), Mathys AG (Bettlach, Switzerland) and Peter Brehm GmbH (Weisendorf, Germany). The assessment of the acetabular defect was based on the standardized planning images (pelvic overview and axial view of the hip joint), computerized preoperative planning as well as computed tomography (CT scans) in individual cases. In all cases, the strategy of the surgical procedures was discussed and scheduled during staff meetings. The final decision was made intraoperatively according to the acetabular defect situation. All patients were treated by experienced orthopedic surgeons in accordance with the internal standard operating procedures (SOP) guidelines. Prior to the revision at hand, most patients had had at least one or more revisions prior to implantation of the abovementioned revision cup (mean 1 ± 0.91). In all cases the preoperative radiological imaging, the operation report and the postoperative radiological imaging were examined. The median age of the patients was 70.5 years (range 45–88 years).

## Results

**Fig. 1:**
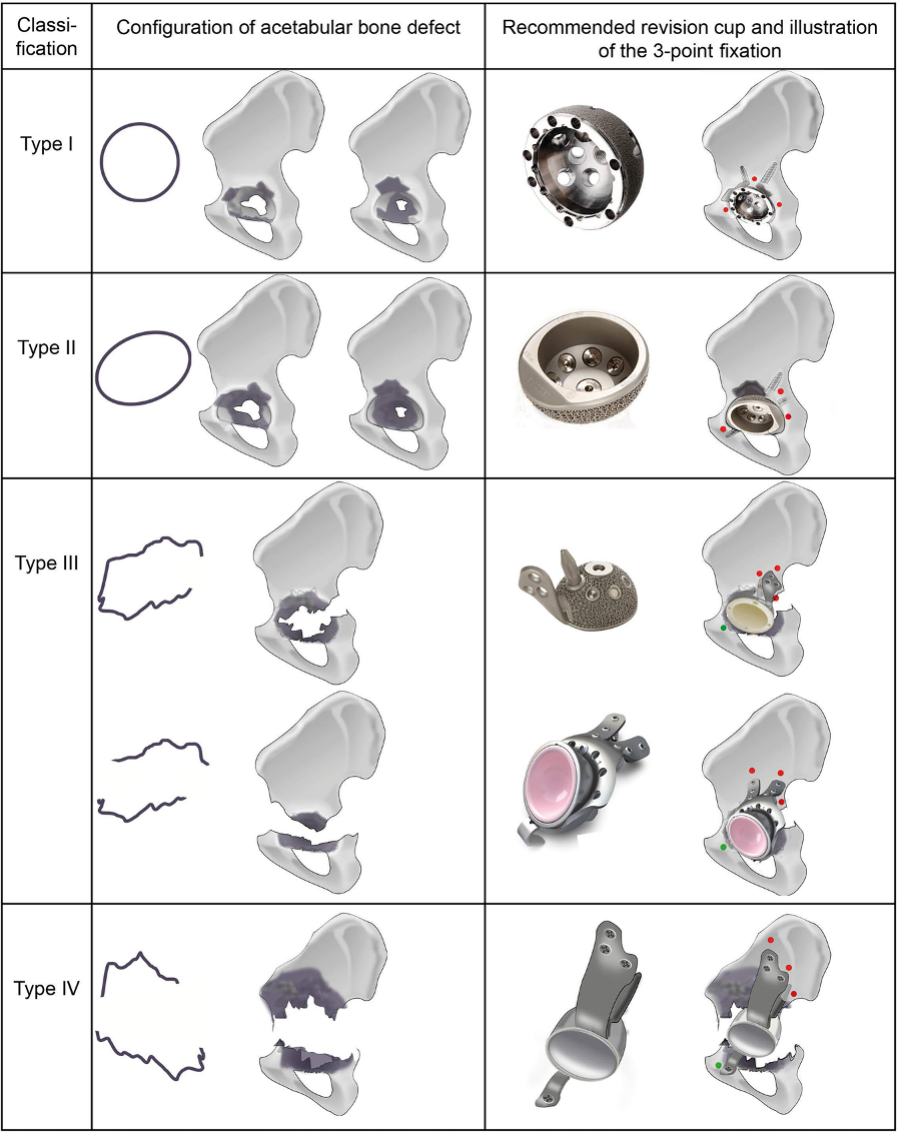
Illustration of acetabular defect classification and management based on 3‑point fixation. *Red points* fixation points. Comment on type III: in cases of implantation of a revision cup with cranial strap and iliac stem, the iliac stem itself is an essential point of fixation. In cases of implantation of the a cup-cage system, the dome screw is an essential point of fixation. *Green points* point of contact with no fixation: type I treated with a spherical multihole cup (DePuy-Synthes); type II treated with an oval cup (AQ Implants, currently AQ Solutions GmbH); type III treated with a revision cup with cranial strap and iliac stem, AQ Implants, currently AQ Solutions GmbH) or with a cup-cage system (Peter Brehm GmbH); type IV treated with a custom-made partial pelvic replacement (AQ Implants, currently AQ Solutions GmbH)

**Fig. 2:**
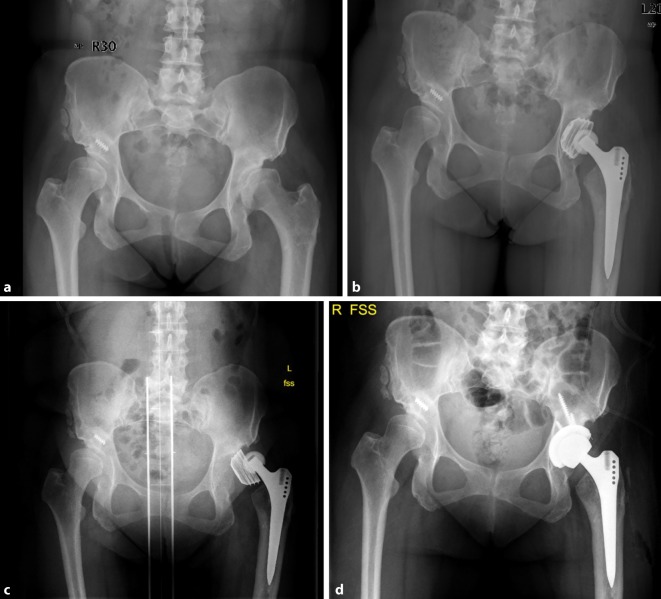
AP X‑rays of the hip (**a**) of a 62-year-old female patient with left-sided dysplasia and coxarthrosis, **b** primary THA in an external hospital, **c** loosening and dislocation of the acetabular cup, **d** revision of the acetabular cup of type I acetabular bone defect using a cementless press-fit hemispherical acetabular cup with additional screw fixation (Mathys AG). (Courtesy of the Department of Diagnostic and Interventional Radiology, University Hospital of Leipzig, all rights reserved)

**Fig. 3:**
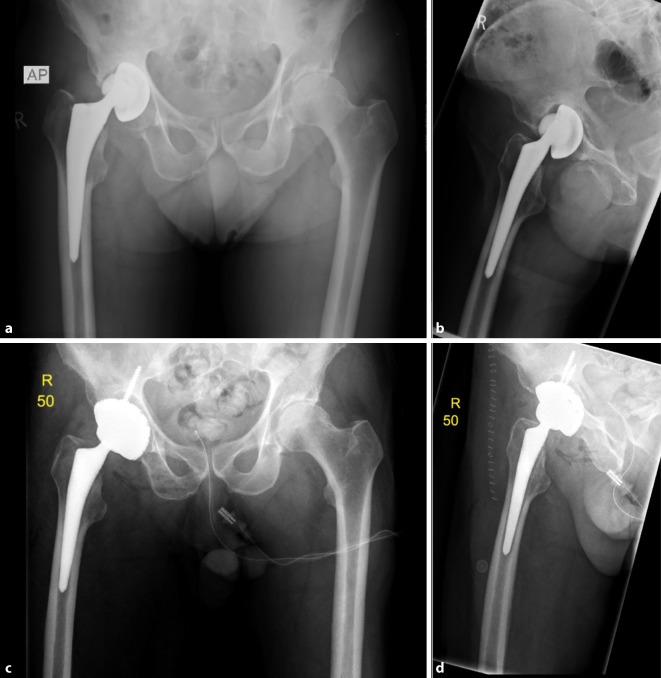
AP X‑ray of the hip (**a**) and axial view (**b**) of a 78-year-old male patient with cup loosening, type II acetabular bone defect; X‑ray of the hip AP (**c**) and axial view (**d**) after treatment with a cementless press-fit acetabular revision cup with additional screw fixation (cranial cup, AQ Implants) (Courtesy of the Department of Diagnostic and Interventional Radiology, University Hospital of Leipzig, all rights reserved)

**Fig. 4:**
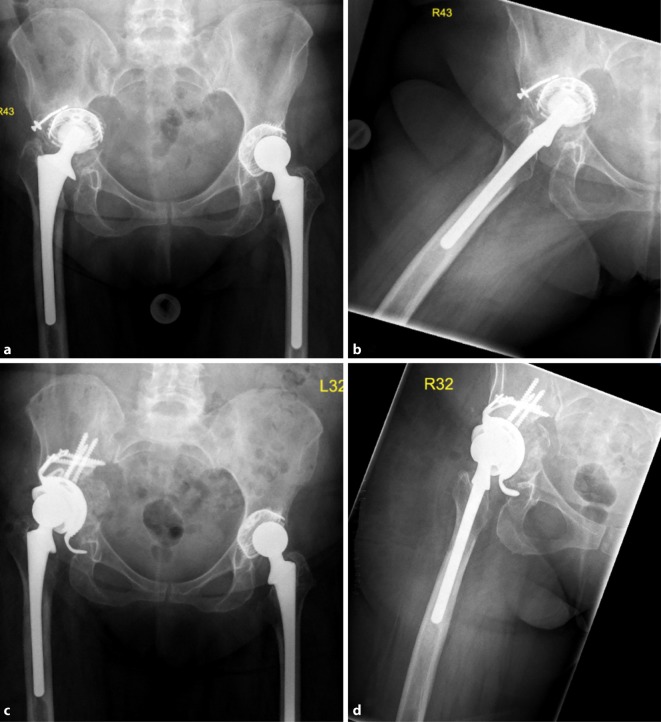
AP X‑ray of the hip (**a**) and axial view (**b**) of an 81-year-old female patient with cup loosening, type III acetabular bone defect; X‑ray of the hip AP (**c**) and axial (**d**) after treatment with an acetabular revision cup-cage component and allogenic cancellous bone (cup-cage, Peter Brehm GmbH) (Courtesy of the Department of Diagnostic and Interventional Radiology, University Hospital of Leipzig, all rights reserved)

**Fig. 5:**
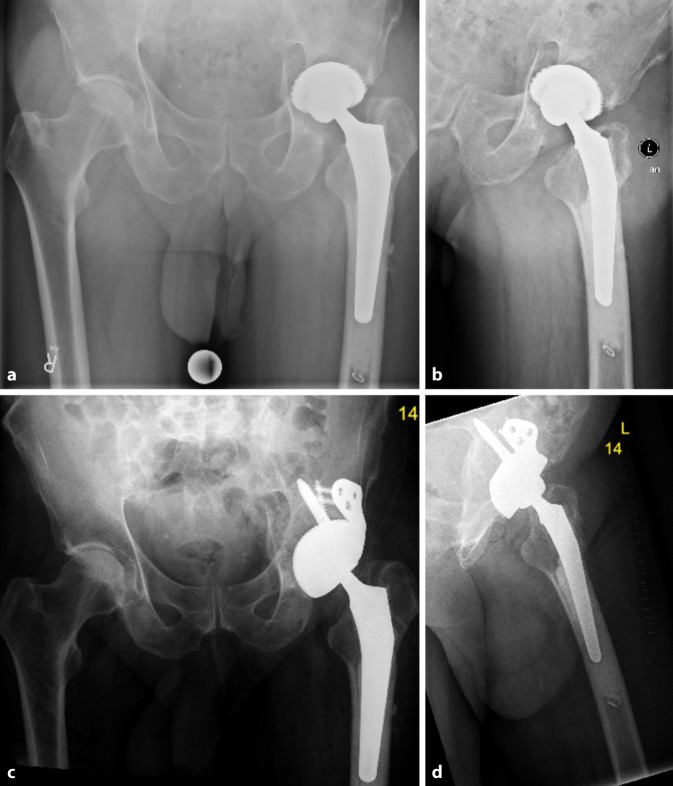
AP X‑ray of the hip (**a**) and axial view (**b**) of a 79-year-old male patient with cup loosening, type III acetabular bone defect; X‑ray of the hip AP (**c**) and axial (**d**) after treatment with an acetabular revision cup (cranial cup with an iliac stem and cranial strap) and allogenic cancellous bone (AQ Implants) (Courtesy of the Department of Diagnostic and Interventional Radiology, University Hospital of Leipzig, all rights reserved)

**Fig. 6:**
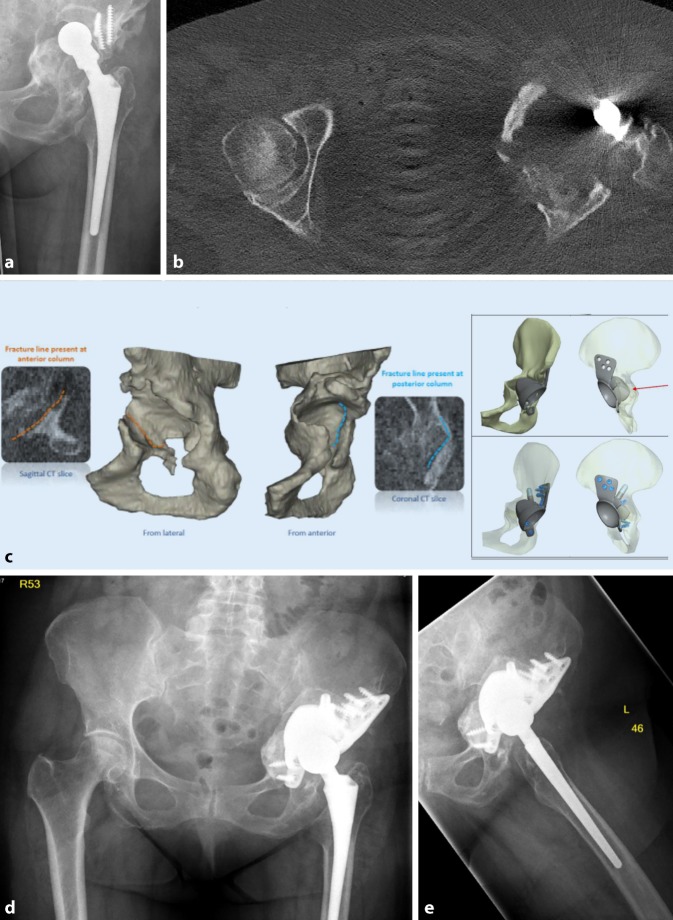
AP X‑ray of the hip (**a**) and CT scan (**b**) of a 77-year-old male patient with cup loosening, type IV acetabular bone defect, **c** imaging show preoperative planning. X‑ray of the hip AP and axial (**d**, **e**) after treatment with partial pelvic replacement (AQ Implants). (Courtesy of the Department of Diagnostic and Interventional Radiology, University Hospital of Leipzig, all rights reserved)

According to the acetabular bony configuration remaining after removing the loosened cup, the revision cup needed was defined and the classification system was developed from this (Tables 1 and 2). Figs. 1, 2, 3, 4, 5 and 6 show examples of each type of this classification.

**Table 1:** Type of revision cup used in this series

Number of patients	Revision cup used in the surgical intervention
76	Cementless hemispherical
36	Cementless oval
46	Cementless acetabular cup with cranial strap ± iliac stem or cup-cage system
2	Custom-made partial pelvic replacement

**Table 2:** Acetabular defect classification and management based on 3‑point fixation

Classification	Acetabular bony configuration	Revision cup needed
Type I	Possible 3‑point fixation within the boundaries of the acetabular wall, hemispherical configuration of the acetabulum	Hemispherical (preferably cementless; cemented only in case of adequate cancellous bone structure and absence of bone defects) ± allogenic cancellous bone
Type II	Possible 3‑point fixation within the boundaries of the acetabular wall, cavitary/oval configuration of the acetabulum	Cementless oval cups or spherical cups with augmentation parts ± allogenic cancellous bone
Type III	Impossible 3‑point fixation within the boundaries of the acetabular wall, cavitary configuration of the acetabulum with severe bone loss or pelvic discontinuity	Cementless acetabular cup with cranial strap ± iliac stem + allogenic cancellous bone or cup-cage system + allogenic cancellous bone
Type IV	Impossible 3‑point fixation within the boundaries of the acetabular wall, pelvic discontinuity with major bone loss and destruction of iliac bone	Custom-made partial pelvic replacement

The key criterion on which the classification is based, is whether or not a 3-point fixation of an acetabular cup within the boundaries of the acetabular wall was possible. The configuration of the bony acetabulum was taken into consideration (Table 2). Minor segmental defects or cup loosening without bone loss can be treated with standard hemispherical acetabular components. Cavitary defects, particularly oval craniolateral defects, can be managed using oval cups or augmentation to restore the acetabular center. In all cases treated with a cementless hemispherical revision cup (76 cases, Table 1), the results of preoperative X‑ray imaging and computerized planning were confirmed by intraoperative findings. A preoperative CT scan was not performed in these cases. In the majority of cases (30 cases out of 36; 83.33%) treated with an oval cementless revision cup (cavitary acetabular defects) the results of preoperative X‑ray imaging and computerized planning were confirmed by intraoperative findings as well (Table 1); however, in 6 cases out of 36 (16.37%) the intraoperative findings showed further defects. Hence, 3‑point fixation within the boundaries of the acetabular wall was no longer possible and these cases were treated by cementless acetabular cup with cranial strap. Again, a preoperative CT scan was not performed in these cases.

The major challenge is dealing with significant bone loss and/or pelvic discontinuity. In all the remaining 48 cases (Table 1) preoperative X‑ray imaging and computerized planning helped to anticipate the magnitude of bone loss. Therefore, CT scans were performed in all of these 48 cases (Table 1) to enable better evaluation of the bony configuration of the acetabulum. Bone loss can be filled with bone grafts and/or treated with the appropriate acetabular component in order to ensure stable anchorage. Several acetabular components are designed to manage bone loss [6–9, 12–14, 16, 22]; however, there are still some cases with pelvic discontinuity with major bone loss and destruction of iliac bone. In these cases, conventional revision cups are no longer suitable and a custom-made partial pelvic replacement must be used.

## Discussion

When grading acetabular defects in revision arthroplasty after THR there is a need for a universal and valid system [17]. Precise classification of acetabular defects has always been a complex problem [7, 17]. Therefore, it is vital to provide a classification system that reliably describes the criteria of the acetabular structure and directly relates to surgical management.

In the literature, several classification systems were proposed and are widely used (Table 3; [1, 3, 5, 11, 12, 19, 20]). When discussing the several classification systems the interobserver (agreement between ≥2 observers) and intraobserver reliability (agreement between the same observer on separate occasions) are highlighted.

**Table 3:** Classification systems for acetabular defects

*1. D’Antonio et al. classification* [3]	
Type I	Segmental deficiency a. Peripheral (superior, anterior or posterior) b. Central (medial wall absent)
Type II	Cavitary a. Peripheral (superior, anterior or posterior) b. Central (medial wall intact)
Type III	Combined
Type IV	Pelvic discontinuity
Type V	Arthrodesis
*2. Engh and Glassman classification* [5]	
Mild	Cavity = hemispherical, cancellous, intact rim = round, strong, intact
Moderate	Cavity = nonhemispherical, sclerotic, perforated rim = round, strong, intact
Severe	Cavity = nonhemispherical, sclerotic, perforated rim = out of round, weak or broken
*3. Gross et al. classification* [11]	
Protrusio	Contained defect with intact rim and columns
Shelf	Defect in rim and cavity with loss of 50% of acetabulum
Acetabular	Defect in one or both columns with 50% loss of acetabulum
*4. Gustilo and Pasternak classification* [12]	
Type I	Minimal cavitary enlargement, loosening of the cement-prosthesis interface
Type II	Thinned, nonperforated wall, loosening of the cement-prosthesis interface
Type III	Local wall defect only a. Anterior b. Posterior c. Superior d. Central
Type IV	Massive and global collapse or defect involving one or both columns
*5. Paprosky et al. classification* [19]	
Type I	Supportive rim with no bone lysis or migration
Type II	Distorted hemisphere with intact supportive columns and 2-cm superomedial or superolateral migration a. Superomedial b. Superolateral (no dome) c. Medial only
Type III	Superior migration 2-cm and severe ischial and medial osteolysis a. Kohler’s line intact, 30–60% of component supported by graft (bone loss: 10 o’clock to 2 o’clock position) b. Kohler’s line not intact, 60% of component supported by graft (bone loss: 9 o’clock to 5 o’clock position)
*6. Saleh et al. classification* [20]	
Type I	No significant bone loss
Type II	Contained loss of bone stock where there is cavitary enlargement of the acetabular cavity but no wall deficiency
Type III	Uncontained loss of bone stock where there is b50% segmental loss of the acetabulum involving anterior or posterior column
Type IV	Uncontained loss of bone stock where there is N50% segmental loss of the acetabulum affecting both anterior or posterior columns (if there is N50% loss of the acetabulum, involving mostly the medial wall but the columns are intact, then this type of defect is considered type II because of the availability of the columns for reconstruction)
Type V	Acetabular defect with uncontained loss of bone stock in association with pelvic discontinuity

The D’Antonio et al. classification system (Table 3) was developed by the American Academy of Orthopedic Surgeons (AAOS) committee on the hip [3]. This classification system undoubtedly gained wide acceptance. It distinguishes between segmental and cavitary defects. The original study involved the evaluation of 83 anteroposterior and lateral radiographs of the hip; however, this classification system did not address reproducibility or validity [17]. Campbell et al. [2] suggested that this system demonstrates poor reliability due to only the originator’s intraobserver κ scores indicating moderate agreement (0.57). For the 3 residents and 3 experts, intraobserver κ scores of 0.37 were achieved by both groups, indicating poor agreement. Interobserver κ scores of 0.37 and 0.16, respectively, also indicated poor agreement for residents and experts [2, 17]. According to Gozzard et al. [10], who evaluated the intraobserver reliability of this classification, the κ indicated poor reliability for consultants (0.37) and registrars (0.60). The interobserver reliability for consultant and registrars was 0.57 and 0.25, respectively; however, this study did not assess the validity [17]. Engh and Glassman [5] provided a simplified version of the AAOS classification (Table 3). This system was developed by evaluating preoperative radiographs; however, intraoperative analysis of the defect types was not addressed. According to Johanson et al. this study did not address reproducibility or validity [17]. The Gross et al. classification system [11] presents a simplified system that focuses on the requirements and specifications for bone graft in the reconstruction. The system was originally developed by intraoperative assessment of 108 hips but was intended for use in assessing preoperative plain radiographs. Although intended to be used preoperatively during radiographic evaluation, the classification described by Gross et al. was originally developed intraoperatively “by visualization, palpation, and use of a trial cup” [11]. Campbell et al. [2] assessed the intraobserver reliability of this system by evaluating 33 hips with AP and oblique radiographs. The originators of the system had found a moderate intraobserver agreement (κ = 0.59); however, when attempting to reproduce these results using 3 residents and 3 experts, intraobserver κ scores of 0.47 were achieved by both groups, indicating poor agreement [17]. Interobserver κ scores for residents and experts were 0.44 and 0.28, respectively [17]. Campbell et al. did not address reproducibility or validity of this system [2, 17]. Gustilo and Pasternak [12] provided a classification system that focused on both the conditions of the remaining bone and the failed endoprosthetic component (Table 3). The system was developed using AP and lateral radiographs of 42 hips. According to the analysis of Johanson et al. [17], the original study did not address reproducibility or validity.

The Paprosky et al. classification system (Table 3) is based on the presence or absence of supporting structures, such as the acetabular rim, superior dome, medial wall, anterior and posterior columns, and the surgeon’s assessment of these structures’ capacity to support the revision prosthesis [19]. This classification system gained wide acceptance [7, 22]. The original classification system was developed by evaluating 147 patients with AP X‑ray imaging, classifying each as type I, II, or III [19]. In 92.5% of the cases preoperative assessment was confirmed by intraoperative findings; however, intraoperative validity did not use weighted κ scores or separate surgeons [17]. Campbell et al. [2] examined the Paprosky et al. system for interobserver and intraobserver reliability. They concluded that the Paprosky et al. system had poor reliability and should be “considered only as a general guide”. Gozzard et al. [10] assessed the reproducibility of the Paprosky et al. system by calculating κ scores for intraobserver agreement and found the system to be unreliable [17].

The classification system proposed by Saleh et al. [20] is based on the evaluation of the anticipated remaining bone stock following removal of the failed implant (Table 3). According to Gozzard et al. and Johanson et al. [10, 17], this is the only classification that was rigorously tested by the original authors and the only one that has been shown to have interobserver reliability. Yet, this classification system did not specify the actual revision options that would be necessary for each type of bone defect [10, 17]. In 1997 Bettin and Katthagen [1] provided the documentation of the German Society for Orthopedics and Traumatology (DGOT) for bone defects in hip revision arthroplasty. According to the authors, the limitation of this classification system lies in the fact that it is based on the evaluation of the preoperative radiographs. The authors stressed the importance of intraoperative evaluation after removal of the loosened cup. This system still has not gained wide application or citation in the literature.

The classification proposed in this study mainly relies on intraoperative findings, which might be regarded as a limitation; however, preoperative X‑ray findings and preoperative computerized planning corresponded to intraoperative findings in all cases of type I and in 83.33% of type II acetabular defects. The evaluation of preoperative radiographs in all remaining cases (type III and IV acetabular defects) led to performing preoperative CT scans to adequately assess the bony configuration of the acetabulum and the magnitude of bone loss. The intraoperative findings confirmed the magnitude of bone loss that were anticipated in preoperative X‑rays and CT scans of all cases with type III and type IV acetabular defects. Overall, intraoperative findings confirmed the preoperative imaging in 154 cases out of 160 (96.25%). This led to the conclusion that the type of acetabular defect according to this classification system can be anticipated by preoperative imaging; however, meticulous preoperative planning based on X‑ray radiographs must be carried out. In addition, a CT scan must be performed as standard preoperative diagnostic procedure whenever type III or type IV defects are anticipated. This classification system is mainly based on the intraoperative assessment of the bony configuration of the acetabulum and the key criterion whether a 3-point fixation of an acetabular cup is technically possible within the boundaries of the acetabular wall. Hence, it directly relates the bony configuration of the acetabulum left for re-implantation with the type of revision acetabular component to be used. This provides correct intraoperative re-evaluation and definitive choice of the necessary implants.

The AAOS classification [3] gained wide acceptance in daily practice. Yet, it was assessed by Gozzard et al. as “poorly reliable” [10].

The present classification system is pragmatic. Types I and II of the classification widely conform to the AAOS classification. The difference lies in the fact that it focuses on treatment and hence on the possibility of 3‑point fixation within the boundaries of the acetabular wall, regardless of the descriptive localization of the bony defect. In contrast to the AAOS classification, type III of this classification system includes those cases in which severe bone loss and/or pelvic discontinuity prevents a 3-point fixation within the boundaries of the acetabular wall. In these cases, additional fixation outside the boundaries of the acetabular wall becomes necessary. Type IV of this classification system introduces an entity with a magnitude of bone loss that exceeds the one described in type IV of the AAOS classification. This newly introduces entity of pelvic discontinuity and major bone loss cannot be adequately treated by conventional revision cups. In this case, a custom-made partial pelvic replacement must be carried out.

In light of the abovementioned classification systems and analyses, it can be stated that this classification system is practicable and pragmatic. It simplifies the assessment of bone defects and is useful to preoperatively evaluate acetabular defects and thus enable proper preparation in terms of surgical approach and necessary implants. Furthermore, this classification system is mainly based on the intraoperative assessment of the bony configuration of the acetabulum and the key criterion whether a 3-point fixation of an acetabular cup is technically possible within the boundaries of the acetabular wall. Hence, it directly relates the bony configuration of the acetabulum left for re-implantation with the type of revision acetabular component to be used. This provides proper intraoperative re-evaluation and definitive choice of the necessary implants; however, this study did not evaluate the reliability of this classification system, which is a limitation. Finally, revision arthroplasty after THR must be performed in centers with sufficient infrastructure and experienced surgeons who can deal with complex situations of acetabular bone defects.

## Conclusion

Many classification systems of acetabular bone defects already exist. Some of them are widely accepted but each has its strengths and limitations. The advantage provided by this new classification system lies in its simplicity and practicability. It directly relates to surgical management options. The proposed classification system is helpful, in particular, for ongoing documentation, for prospective investigations and for the handling of scientific analysis.

## Abbreviations

AAOS: American Academy of Orthopedic Surgeons
AP: Anteroposterior
CT: Computed tomography
DGOT: German Society for Orthopedics and Traumatology
EPRD: German Endoprosthesis Register
ICD10: International Classification of Diseases 10
OECD: Organization of Economic Cooperation and Development
SOP: Standard operating procedure
THA: Total hip arthroplasty
THR: Total hip replacement
